# Quantifying CrossFit^®^: Potential solutions for monitoring multimodal workloads and identifying training targets

**DOI:** 10.3389/fspor.2022.949429

**Published:** 2022-10-14

**Authors:** Gerald T. Mangine, Tucker R. Seay

**Affiliations:** Exercise Science and Sport Management, Kennesaw State University, Kennesaw, GA, United States

**Keywords:** high-intensity functional training (HIFT), workout pacing, overtraining, athletes, competition

## Abstract

The design of high-intensity functional training (HIFT; e. g., CrossFit^®^) workouts and targeted physiological trait(s) vary on any given training day, week, or cycle. Daily workouts are typically comprised of different modality and exercise combinations that are prescribed across a wide range of intensities and durations. The only consistent aspect appears to be the common instruction to maximize effort and workout density by either completing “as many repetitions as possible” within a time limit (e.g., AMRAP, Tabata) or a list of exercises as quickly as possible. However, because effort can vary within and across workouts, the impact on an athlete's physiology may also vary daily. Programming that fails to account for this variation or consider how targeted physiological systems interrelate may lead to overuse, maladaptation, or injury. Athletes may proactively monitor for negative training responses, but any observed response must be tied to a quantifiable workload before meaningful changes (to programming) are possible. Though traditional methods exist for quantifying the resistance training loads, gymnastic movements, and cardiorespiratory modalities (e.g., cycling running) that might appear in a typical HIFT workout, those methods are not uniform, and their meaning will vary based on a specific exercise's placement within a HIFT workout. To objectively quantify HIFT workloads, the calculation must overcome differences in measurement standards used for each modality, be able to account for a component's placement within the workout and be useful regardless of how a workout is commonly scored (e.g., repetitions completed vs. time-to-completion) so that comparisons between workouts are possible. This review paper discusses necessary considerations for quantifying various HIFT workout components and structures, and then details the advantages and shortcomings of different methods used in practice and the scientific literature. Methods typically used in practice range from being excessively tedious and not conducive for making comparisons within or across workouts, to being overly simplistic, based on faulty assumptions, and inaccurate. Meanwhile, only a few HIFT-related studies have attempted to report relevant workloads and have predominantly relied on converting component and workout performance into a rate (i.e., repetitions per minute or second). Repetition completion rate may be easily and accurately tracked and allows for intra- and inter-workout comparisons. Athletes, coaches, and sports scientists are encouraged to adopt this method and potentially pair it with technology (e.g., linear position transducers) to quantify HIFT workloads. Consistent adoption of such methods would enable more precise programming alterations, and it would allow fair comparisons to be made between existing and future research.

## Introduction

High-intensity function training is a term used to describe both a training strategy and sport that incorporates a variety of functional, multimodal movements into workouts meant to be performed at a relatively high-intensity for the purpose of improving (or testing) general physical preparedness (1, 2). Although this definition encompasses a variety of specific programming models, CrossFit^®^ (CF) training may be the most popular example (1). The CF training strategy aims to avoid forming any linear structure to its daily, weekly, and monthly programming, and instead, attempts to constantly vary the stimulus to promote simultaneous and generalized improvements in fitness (2). CF competition events mirror this endeavor by variably programming workouts to challenge aptitude in any combination of strength, endurance, power, and skill across multiple modalities (3, 4). Thus, it is not surprising that most physiological traits tested in CF athletes (5–11), as well as a variety of training and past-competition experiences (6, 11, 12), have been related to performance with no consensus as to which is most important. However, this presents a potential trap for those who do not possess the expertise needed to correctly implement the training model, as well as to those who wish to excel in the sport.

Although athletes and coaches may sequence CF workouts in a coordinated, progressive, and periodized manner, the original intention was to do this in a more randomized and variable fashion to elicit simultaneous and generalized improvements across all relevant areas of fitness (2); like non-linear programming (13). Without a thorough understanding (or consideration) of the relationships between programming variables and their effect on human physiology, however, it is quite easy to inadvertently overemphasize specific and/or competing physiological systems and muscle groups during training (13, 14). Likewise, the lack of any hierarchical importance among CF-relevant physiological traits may tempt athletes to do this more intentionally (i.e., attempting to simultaneously train for everything). Indeed, several combinations exist among the 10 fitness domains cited by the Level 1 Training Guide (2) where a pair of training targets should be sequenced appropriately (e.g., sports-specific skill and muscular endurance, maximal strength and aerobic endurance) to avoid activating opposing physiological adaptation mechanisms (14). At minimum, the accumulated fatigue associated with improper sequencing or integration could result in blunted adaptations, and in worse case, overtraining and injury. That said, a growing body of evidence suggests that CF training does not appear to present any greater injury risk than more traditional training modalities and sports (15–18). Regular training on ≥ 3 days per week for more than 1 year (15) under the supervision of expert coaching (18) appears to further reduce these risks. Unfortunately, the actual training habits among various CF subpopulations are not well-documented, and no study has attempted to compare training tactics among CF coaches of various levels of expertise. Nevertheless, it might be hypothesized that careful manipulation of programming variables and monitoring workload are essential to competitive success and training safety.

Compared to more traditional modalities and sports, some of which have been around for centuries, our understanding of CF training (founded in 2000) and its associated sport are in their infancy. From 2012 to 2022, the body of CF-related literature has grown from <10 to over 250 peer-reviewed articles spread across a variety of topics. However, these studies have varied in reporting structure and terminology, scientific rigor, populations studied, variables considered, and most importantly, their context. For instance, studies examining injury risks are based on data obtained from surveys requesting generalized descriptions of training habits among healthy adults (15–18). The volume or sequencing of work completed throughout training were not quantified overall, nor with respect to specific physiological systems and muscle groups. In fact, no CF-related study has provided more than a list of programmed exercises and summary estimates of training frequency, intensity, and duration. Without these, reproducibility of any CF training intervention is nearly impossible, and thus, prohibits any definitive conclusions from being made at this time. In part, the lack of uniformity and well-described context is the consequence of there being no simple, universal method for quantifying CF workloads.

Within more traditional sports' settings, strength training and conditioning exercises are often prescribed to be performed separately. This allows workloads (e.g., average intensity, volume loads, etc.) to be easily calculated for both individual exercises, entire workouts, and training weeks. Further, because most periodization structures used within these settings do not involve drastic daily or weekly changes in exercise/drill prescription and programming (13, 19), following their progress is a relatively simple task. Coaches may also use a variety of tools ranging in sophistication, cost, and focus (e.g., heart rate and heart rate variability, RPE and other subjective perception surveys, global positioning systems and accelerometers, velocity-based assessments like vertical jump performance) to tie completed workloads to a monitored training response (20, 21). They can respond to a series of negative observations by adjusting associated workloads (e.g., reduce resistance training volume loads, running distances, rest intervals) to alleviate the stress. This idea of monitoring training responses is also relevant to CF training, and some have recommended pairing RPE (22) with heart rate variability (23) for this purpose. However, while these tools provide insight into the resultant training stress, they are of little value if they cannot be tied to a cause (i.e., the workload).

A multitude of resistance training, gymnastic, or cardiorespiratory (referred to as “monostructural”) exercises and modalities may simultaneously be programmed into a single CF workout (1, 2). The different methods traditionally used to quantify each are not adequate for quantifying CF workloads. The most obvious reason is because workloads for each modality are calculated differently and expressed in different units (e.g., resistance training volume loads vs. running distances) (24–27). Simplifying each calculation into a single numerical value to quantify workload would not be possible whenever a CF workout incorporated multiple modalities, which is quite common. Indeed, out of the 48 unique workouts programmed throughout the history of the CrossFit^®^ Open (CFO), the opening stage of the annual CF competition, 90% incorporated at least two exercises where workload would be quantified using different traditional calculation methods and units (3, 28). Even if it were conceded that describing CF workloads with multiple values was acceptable, doing so would still fail to account for the impact of exercise placement and the workout's structural design to the overall training stress.

Unlike more traditional training strategies, the definition of CF allows for daily variation in workout exercise composition, structural complexity, and expected volume and density (3, 4). Each day, the specific combination of these programming traits may differ drastically, which in turn, may alter the relative intensity and difficulty of each prescribed component. The same component present in two different workouts might provide a completely different training stimulus because of differences in its placement within each workout, the presence of other exercises, and overall workout structure. For example, the benchmark workout “Cindy” requires trainees to repeat a circuit of 5 pull-ups, 10 push-ups, and 15 bodyweight squats as many times as possible in 20 min (29, 30). There are no minimum or maximum limits placed on repetitions completed. Trainees are simply encouraged to maximize their workload within the set time limit, making the relative intensity of these three exercises and the trainee's effort the primary contributors to the overall workout stress. In contrast, the hero workout “Murph” consists of the same three exercises programmed at a volume that would equate to 20 rounds of “Cindy”, but sandwiched between two, 1-mile runs (10, 30). If completed as prescribed (i.e., Rx), trainees would run one mile, complete 100 pull-ups, then 200 push-ups, then 300 bodyweight squats, before running the second mile, all while wearing a weighted vest. Trainees also have the option of eliminating the vest and/or partitioning the workout components to best fit their level of fitness. In either case, the goal is to complete the assigned work as quickly as possible. If one were to compare the “Cindy” performance to performance in the calisthenics portion of “Murph” using traditional quantification methods, some might conclude that 20 rounds of “Cindy” was an average effort (31). However, doing so would fail to acknowledge the impact of running the first mile on the relative intensity of the calisthenics exercises, as well as the possibility of the effort being modified to manage fatigue prior to the second mile run. Carreker et al. (10) reported “Murph” times that were 2–3 times (36.56–54.21 min) the duration of “Cindy,” with an average of 25.5 ± 3.7 min being spent on the calisthenic components. The additional ~5.5 min clearly suggest different pacing strategies will be used for “Cindy” and “Murph,” and each would be representative of different percentage of the entire workout stress. Percentages that could be further altered by different partitioning strategies. Thus, limiting between- or within-workout comparisons to modalities with matching workload calculations would not adequately describe workload.

Traditional quantification methods might provide objective information but provide little value for contextualizing the contribution of each workout component to the overall training stress or to other workouts of similar or completely different design. The addition (or subtraction) of one or more different exercise modalities, their placement within a workout, different workout structures, as well as how these may differ drastically from day-to-day, are all important considerations when attempting to understand (and make comparisons between) each workout's impact within a training cycle. The accounting process should be able to equate, for instance, a workout that requires 10 rounds of barbell thrusters and double-unders to a 15-min workout that includes wall ball shots and rowing, or when one workout contains two repeated exercises whereas another consists of 10 exercise that are each performed once. It should allow athletes and coaches to more precisely scale or progress training workloads based on monitored training responses. Meanwhile, with less than a handful of specific workouts being represented more than once across all studies examining physiological responses to CF or high-intensity functional training, the lack of a unifying method for quantifying CF workloads makes it extremely difficult to form any generalized conclusions. Having a method that would allow sports scientists to make fair comparisons within and across studies would enable evidence collation and facilitate such recommendations. Therefore, the purpose of this paper is to discuss potential methods for quantifying CF workout performance and future research directions.

## Workout components, structure, and scoring

### Exercise selection and quantification

By definition, CF workouts may include, but are not limited to, any exercise that falls into one of three main categories: (1) weightlifting, (2) gymnastics, and (3) monostructural (1, 2). Here, weightlifting refers to both its traditional definition (i.e., Olympic Weightlifting; snatch, clean, and jerk variations), as well as power lifts (e.g., squats, deadlifts, and presses) and other traditional resistance training exercises that utilize external loads (i.e., kettlebells, dumbbells, and medicine balls). Gymnastics refer to basic (e.g., sit-ups, push-ups, and burpees) and complex (e.g., muscle-ups and handstand walking) calisthenic movement patterns and require the trainee to move about or over an obstacle (e.g., pull-up bar, gymnastic rings, boxes, and ropes). Monostructural exercises are basic movement patterns performed continuously in cyclical fashion and are those that commonly appear in traditional cardiovascular/aerobic training (e.g., rowing, running, biking, swimming, or skiing). All three exercise categories may appear in any CF training workout though options are less extensive during competition, particularly during the CFO. The CFO is an international competition that was first introduced in 2011, and it allowed athletes to complete a series of 3–6 workouts at their local training facility over the course of 3–5 weeks (3) and earn the right to advance on to later stages. However, because of difficulties with standardizing certain modalities (e.g., running courses, swimming) and equipment across locations, CFO workouts tend to include exercises and modalities that are available at nearly any affiliated location (3, 4). The selection expands toward all possibilities as the competition approaches the final round (i.e., the Games^TM^).

Regardless of whether an exercise appears within a training or competition workout, its individual prescription is conducted in the same manner and this manner can vary across, and sometimes within, exercise categories. For instance, weightlifting exercises primarily utilize traditional methods for quantifying intensity loads (i.e., absolute load, relative or percentage-based, and repetition-maximum [RM]) and volume (i.e., sets and repetitions), which makes volume load a simple calculation (24–26). Mechanical work and total work might also be calculated from load displacement (25), though not as easily without standardizing range of motion on each repetition or utilizing monitoring equipment (25, 26). Calculating work is further complicated when a load deviates from a linear trajectory (32, 33), as is possible during various Olympic lifts and kettlebell exercises. Likewise, work estimates are difficult when the exercise involves throwing and catching a projectile, such as the wall ball shot exercise (pairs a medicine ball front squat with a shot to a target). Even with standardized squat depth criteria, target distance, and medicine ball mass for this exercise (3), it is virtually impossible for athletes to squat the same depth or shoot the medicine ball to the same target spot on each repetition. Either might lead to differences in the amount of work being performed on each repetition, and an athlete's height and stance width only further complicate this by altering squat range of motion (34) and shot distance to target. Overall, however, using traditional methods for quantifying resistance training intensities and workloads within the context of a CF workout will always be problematic. The sheer number of possible exercises appearing within a single training cycle alone would make relating assigned loads to a 1-RM or RM load a pointless and impossible task. Even if this were possible, the load's contribution to the overall training stress would be dependent on the exercise's placement within the workout structure.

Gymnastic movements present an even more difficult challenge for monitoring performance. Though several can be quantified by volume load, where body mass is the load, their contribution to the overall training stress is also dependent on workout placement, as well as the trainee's proficiency in performing the movement. Variations in technique can drastically affect activated musculature and work performed. For instance, effective utilization of lower-body technique is thought to impact work performed by the upper-body musculature and the accumulation of fatigue during rope climbing (35). Because of this, it is possible for skilled athletes to outperform those who lack this skill, even when they possess less strength or endurance. Similarly, utilizing a kipping or butterfly motion (i.e., swaying to create hollow and arched body positions) builds momentum that assists the concentric phase of a pull-up. Compared to a strict pull-up, Williamson and Price (36) noted reduced biceps brachii and latissimus dorsi activation and increased rectus abdominus, gluteus maximus, and rectus femoris activation during kipping and butterfly pull-ups, which might help delay fatigue. It is logical to assume that a kipping motion would also assist the concentric phase of the handstand push-up exercise, but evidence is limited to one study examining muscle activation differences between push-ups and the shoulder press (37) and a review paper discussing technical progressions in the strict handstand push-up (38). Nevertheless, the implications of utilizing momentum to facilitate an exercise and delay fatigue extends beyond performance quantification. Theoretically, using a kip creates a more rapid stretch between the eccentric and concentric phases, like that which occurs during plyometric exercise. Such movements enhance the mechanical stress placed on activated tendons and muscle, stress that has the potential of being too great under fatigued conditions and lead to failure or strain (39, 40). Conversely, additional strain, effort, and work might occur when the athlete lacks skill and/or efficiency in an exercise (40). Thus, the caveat to monitoring volume loads and work completed during gymnastic movements is that technique (or lack of technical skill) must be considered, and this may change (intentionally or unintentionally) within and across sets.

Technique is also a worthy consideration with monostructural movements. Except for jumping rope (repetitions), which could also be placed within the gymnastics category, CF typically limits prescription to distance (all modalities) or calories (rowing, cycling, and skiing) (3). Repetitions, distance, and calories serve as adequate metrics of volume and are easily tracked, but they provide little insight into the relative training stress. This is mainly determined by the oxygen cost associated with the athlete's pace relative to their aerobic capacity (27). While pace may be derived by factoring in duration, aerobic capacity is not as easily obtained and subject to variability across each relevant modality (41). Like resistance training exercises, finding a trainee's aerobic capacity in each possible modality, and relating those values to workout performance, would be extremely time-consuming and probably unnecessary. The oxygen cost of each unique effort [e.g., each time a trainees runs during the benchmark workout “Helen” (42), which consists of three rounds of a 400-m run, 21 kettlebell swings, and 12 pull-ups] would vary across the workout, and need to be viewed in light of its placement within the overall workout structure. With it already being well-established that CF workouts will typically produce post-exercise heart rate and lactate values (43–50) that are consistent with vigorous activity (51, 52), quantifying the progression of oxygen cost over the course of a workout is appealing and applicable to all exercise types. However, current methods for its collection (e.g., portable gas analysis systems) are too invasive to have any practical value within a CF setting, and the availability and cost of associated equipment only make their adoption more unlikely. Even if oxygen cost could be determined without impeding exercise, that value would still be complicated by a variety of other factors (e.g., technical efficiency, relative difficulty of other exercise modalities, accumulated fatigue) known to influence the oxygen cost of the trainee's chosen pace (27). Ultimately, the trainee's pace within the context of workout's prescription is an easier and universal calculation that can be made relative to other workout components and still provide an indication of effort.

In short, the collection of possible CF workout designs makes the quantification of relative workload a tedious endeavor. For this reason, some have avoided quantifying workload in favor of simply reporting the frequency in which various exercises appear during training (stated as a percentage of appearances among all assigned workouts) (53). At best, this might help the athlete and coach identify movement patterns that are overused during training, but without any insight on work, volume load, or context. No, the relative contribution of each exercise is needed and fortunately, there are inherent aspects about CF workout structures that only require a small amount of manipulation to provide insight.

### Workout structure and quantification

Exercises placed within a CF workout may receive specific or undefined prescription (1, 2). Absolute, relative (e.g., percentage-based), or repetition-maximum (RM) loads may be assigned for a specific or indefinite number of repetitions (or durations), sets, or rounds, and prescription may vary within and across each exercise and set/round. Except for competition, trainees also have the option to scale prescription to match their capabilities, strength, and fitness (1, 2). This also includes scaling rest intervals between exercises, sets, and rounds. Rest in a CF workout is rarely defined and is instead, autoregulated by the trainee (i.e., rest “as needed”). It is this latter aspect that potentially shifts a workout's difficulty and required effort away from the prescription of its individual components toward its structure and supplementary instructions.

Between warm-ups and cool-downs, training sessions may consist of one or more “workouts,” and these may emphasize strength-power development, sport-specific skill practice or acquisition, or metabolic conditioning” (METCON) (2). The emphasis is derived from the supplementary instructions attached to the workout. For instance, although a specific amount of time within a session may be allotted for strength-power and skill-based sessions, completing work as fast as possible is not usually the emphasis. Rather, strength-power workouts closely resemble traditional resistance training, and trainees are either instructed to execute lifts with RM loads and either rest for a pre-defined interval or “*as needed*.” Trainees must still be considerate of the allotted time but maintaining technical standards (i.e., by using appropriate loads and not rushing through sets) is either implied or outright stated “*for quality*.” There is little difficulty in quantifying these workouts. In contrast, the need to quantify skill-based workouts depends on the trainee's aptitude in the “skill” being practiced. Practicing gymnastics skills (e.g., ring muscle-ups) might involve a series of progression drills meant to facilitate motor learning for a novice trainee, or more advanced drills for the experienced athlete. Although monitoring the effort and rest between drill attempts is advisable, particularly when the trainee cannot execute a drill efficiently, a present coach and a “*for quality*” instruction may help to limit the workload and need for precise quantification. This need becomes greater when the “skill” being practiced involves a conditioning aspect, for example:

- executing a complex movement immediately following a fatiguing event
- transitioning between specific exercises
- barbell cycling
- continuous and consistent effort

METCON workouts predominantly emphasize maximizing workout density (i.e., more work in less time) or sustaining effort (1, 2), which entail a few common strategies. METCON's are typically devised as a circuit to be completed for time or repetitions. In either case, exercises are drawn from any of the three exercise categories and given specific loading (when applicable), repetition/duration, and set/round prescription. The order of exercises is also explicitly stated, though, there are times when the trainee may be free to self-select an appropriate ordering strategy. Trainees are either tasked with completing the assigned work as quickly as possible and scored by their time to completion (TTC), or they must repeat the circuit for “as many rounds/repetitions as possible” (AMRAP) within a specified time limit. Greater workload density is accomplished by either completing a fixed amount of work in the shortest amount of time (i.e., TTC structure) or by maximizing work completed within a fixed time (i.e., AMRAP structure). Sustained effort is also implied by both strategies, as finding a pace that minimizes inactivity (e.g., rest breaks and transitions) would theoretically maximize density.

There are also some exceptions to these two structures, but they are less common and have not appeared in official CF competition (3). One example is the Tabata protocol (54), named after the author of a classic investigation comparing continuous and high-intensity efforts on aerobic and anaerobic outcomes (55). It is an AMRAP subcategory where trainees are tasked with completing as many repetitions as possible of an exercise within 20 s. They are then given 10 s of rest before repeating the effort for a total of eight rounds (4 min). A more extended format assigns an amount of work to be completed “*every minute on the minute*” (EMOM) for a set duration (e.g., 15 burpees every minute for 15 min). In this latter example, the longer active period allows trainees to strategize by either completing assigned work as quickly as possible and resting the remainder of the minute or spreading the work evenly throughout the entire interval. The EMOM structure can be further manipulated by shortening or lengthening the interval periods to target a specific work rate (e.g., E2MOM, every 2 min on the minute), or by progressively increasing required work after a set number of intervals (e.g., “Death by…” workouts, where repetitions are added on each minute) to encourage a progressively faster pace. In any case, there are several options for scoring Tabata- and EMOM-style workouts. A score might be based on the slowest TTC across all intervals, the least number of repetitions across all intervals, the total number of repetitions across all intervals, total workout duration, or no score at all. There are also instances, particularly with EMOM's, where the workout concludes if the trainee cannot complete the workload within the interval period.

## Quantifying and comparing workout strategies

### Repetition completion rate

In competition, because a winner must be found, workouts are structured so that the resultant score distinguishes performance. The specific workout structure influences whether a score is possible and how that score may be recorded (i.e., TTC or repetitions). The extremely short work-to-rest ratio of Tabata-style workouts makes accurate scoring difficult, while the shorter overall duration may not be long enough to distinguish performance among athletes of similar skill and fitness. Likewise, the possibility of several athletes tying over the course of an EMOM limits the appropriateness of this structure in competition. Thus, most training and all competition workouts are structured using the AMRAP and TTC formats; though at times, workouts may simply require the athlete to perform a one-repetition maximum (1-RM) or RM, and these are scored by load lifted (3, 4).

Scoring for maximal strength and AMRAP workouts is straightforward: the load lifted, or repetitions completed within the time limit determine the athlete's ranking, respectively. In contrast, TTC workouts are more complicated. A time limit is usually attached to TTC workouts that acts as a scoring threshold. Athletes who complete all assigned work within the time limit are scored as TTC (in minutes and seconds), while those who do not are credited with the work (i.e., repetitions) they completed before time expired. Accurate comparisons between athletes or performances are more difficult when a workout can be scored in two different ways. Although those who complete the workout before time expired clearly outperform those who do not, the exact difference is unclear. One solution is to convert a workout's score into a repetition completion rate (e.g., repetitions·minute^−1^). Doing so places both sets of athletes on the same scale with better performances being identified by a higher rate. This strategy may also be employed to quantify and make limited comparisons between TTC and AMRAP workout performances.

Table 1 provides the prescription, average score (of men and women ranking within the 50th percentile) (56), and the score converted into a rate for the five benchmark workouts that users may post to their online profile at the official CF website (42): “Grace,” “Fran,” “Helen,” “Fight Gone Bad (FGB),” and “Filthy-50 (F50).” These are referred to as benchmark workouts because of their notoriety and standard prescription. In this example, the traditional score and repetition completion rate are of similar value when comparing men to women but not between workouts. Average scores for the four timed workouts are all different, and the fifth workout (i.e., FGB) is scored by repetitions completed. Making comparisons based on traditional scoring would indicate that all five workouts are generally different. However, the rate calculation reveals “Grace,” not FGB, to be the outlier among these five workouts. “Grace” and “Fran” are anecdotally regarded as “sprint” workouts due to their short, expected durations and low number of prescribed repetitions. Despite that, the repetition completion rate of “Grace” was half of that seen in the other workouts. Previous studies have noted differences in the physiological predictors of “Fran” and “Grace” (5, 8, 57) and pacing may be relevant.

**Table 1:** Workout structure and average repetition completion rate for five benchmark workouts.

**Workout**	**Structure**	**Workout**	**Score**	**Rate**
*Grace*	Complete one round of:	30 clean and jerks (61.2 kg)	M: 3:00	10.0
TTC (minutes)			F: 3:33	8.5
*Fran* TTC (minutes)	3-round circuit performing 21 repetitions (round 1), 15 repetitions (round 2), and nine repetitions (round 3) of:	Thrusters (43.1 kg)	M: 4:10	21.6
		Pull-ups	F: 5:31	16.3
*Helen*	Complete three rounds of:	400 m run*	M: 11:11	19.8
TTC (minutes)		21 Kettlebell swings (24.0 kg)		
		12 Pull-ups	F: 10:21	21.4
		*Example awards 1 repetition every 10 m		
*Fight Gone Bad*	Three rounds of five 1-min stations scored by points	Wall-ball (9.1 kg, 3 m target)	M: 335	19.7
17-min AMRAP (repetitions)		Sumo deadlift high-pull (34.0 kg)		
		Box jump (0.5 m box)		
		Push-press (34.0 kg)	F: 292	17.2
		Calorie row		
		1-min rest		
*Filthy 50*	50 repetitions of:	Box jumps (0.6 m box)	M: 24:22	20.5
TTC (minutes)		Jumping pull-ups		
		Kettlebell swings (16.4 kg)		
		Walking lunges		
		Knees-to-elbows		
		Push press (20.4 kg)	F: 27:20	18.3
		Back extensions		
		Wall-ball shots (9.1 kg, 3 m target)		
		Burpees		
		Double-unders		

Although calculating a workout's repetition completion rate is useful for making fast comparisons between athletes and workouts, there are still notable shortcomings:

- It represents the average pace used over an entire workout. It does not account for the natural or strategic variations in pacing that may occur between exercises, at different stages of the workout, or both.
- A higher rate may be indicative of either greater fitness or an easier workout. Neither is clearly identified because the workout's (and each component's) relative intensity and complexity are not distinguished.
- Bias may be introduced whenever a workout contains components that are not traditionally quantified by repetitions completed. For instance, a specific definition was used to convert the 400-m sprint portion of “Helen” into repetitions completed. Modifying or eliminating this definition could either raise or lower the calculated rate.
- Many of the factors that contribute to the total workout stress (e.g., number of exercises, total volume load, total workout duration) are minimized or masked by converting the score into a rate.

Coaches, athletes, and sports scientists must understand and acknowledge these limitations before making any conclusions about a performance based on an overall repetition completion rate.

### Work and power calculations

Ideally, quantifying the individual contribution of each component within a workout would provide greater insight into the overall training stress. The Level 1 Training Guide (2) provides an example of estimating workload and power output for the benchmark workout “Fran,” which is repeated here in Table 2 for a 95-kg athlete. The example approximates work by multiplying force and concentric vertical displacement. Force is defined by mass (i.e., the athlete's body mass for pull-ups and the athlete's body mass + barbell load for thrusters) and distance is based on the trainee's height and limb lengths. The sum of work calculated for all repetitions of each exercise is divided by TTC to estimate power output. Although this calculation is meant to be an approximation and the idea has merit, it suffers from several inaccuracies that limit its value. First, derived work and power may only be considered external (or resultant) (58), as they do not represent the energy used for muscle actions or that which is lost in non-propulsive directions. This is less important for exercises that follow a predominantly linear path along the vertical axis (e.g., thrusters) than it is for those that may utilize technical variations to facilitate vertical momentum (e.g., kipping and butterfly pull-ups involve sagittal deviations to create momentum). When technique is a factor, as is the case in the latter scenario, the trainee's skill in their preferred technique influences muscle activity (36) and the work performed. The work calculation is also based on an incorrect approximation of force (i.e., mass times acceleration) (59). The example correctly addresses mass but not acceleration, and it assumes that concentric vertical displacement is constant. When the mass of an object is the only consideration, the calculated force is only representative of that which is required for the object to remain stationary against the force of gravity (59). This results in an underestimation of force produced during any repetition. Overestimation is also possible, especially when an exercise does not involve 100% of the individual's body mass (e.g., a portion of the lower limb is stationary during a squat). Meanwhile, variations in concentric vertical displacement (e.g., squat depth, arm extension) are likely to occur and alter the amount of force expressed on each repetition. These issues limit any calculated value from being an accurate estimate of work, which also impacts the accuracy of the power output calculation. Ultimately, the power calculation seems more complicated than simply finding repetition completion rate, and without providing additional insight (theoretical or documented in research). Still, an argument can be made that it is the first instance where the contribution of individual workout components to the overall workload were considered.

**Table 2:** Workload and power output estimate for the benchmark workout “Fran.”

	**Force**		**Distance**	**Work**	**Repetitions**	**Total work**
	**kg**	**N**	**m**	**J**	**#**	**J**
Pull-ups	95	932	0.55	512	45	23,058
Thruster (athlete)	95	932	0.83	773	45	34,796
Thruster (barbell)	43	422	1.38	582	45	26,187
Total						84,041
Completion time (sec)						222
Power output (W)						379

### Workout component pacing

Another approach is to utilize a coach or judge (60) or a video recording (61–64) to document start and end times for each exercise, set/round, break, and transition, as well as the occurrence of failed repetitions (i.e., “No-Reps”), within an entire workout. This approach was initially proposed in a pilot study that aimed at relating different pacing strategies to 2016 CFO performance (60). In that study, the authors first calculated repetition rate for each exercise and round (i.e., repetition rate for all exercises within a single round), and then related the average, slowest, and fastest exercise/round repetition rates to the overall workout completion rate. The average was meant to reflect the typical pace employed for each exercise or round throughout the workout, whereas the fastest and slowest rates reflected maximum and minimum efforts, respectively. Additionally, the general trend in how repetition rate changed over the workout was quantified by slope. A positive slope meant pace got faster as the workout progressed, a negative slope meant pace got slower, and values close to zero implied a consistent pace. These calculations (i.e., average, shortest, longest, and slope) were also applied to rest times between exercises and rounds over the entire workout. More recently, this approach was updated for a series of presentations on the 2020 CFO workouts to add a distinction between exercise/round transitions and rest breaks, as well as to introduce standard deviation (SD) (61–64). Like slope, SD described pacing variability but more succinctly; a lower value meant more consistency whereas a higher value implied greater variability. An example of this may be viewed in Table 3.

**Table 3:** Repetition and round completion rate for thrusters and pull-ups in the benchmark workout “Fran.”

**Stage**			**Time**	**Rate**	**Summary**	
			**sec**	**reps·sec^−1^**	
Round 1 (21 repetitions)	Start at 0:00					
		Thrusters × 21	43	0.49	Thrusters repetition rate	
	0:43				Average	0.50
		Transition	8		Fastest	0.50
	0:51				Slowest	0.49
		Butterfly pull-ups × 12	15	0.80	Slope	0.01
	1:06				SD	0.01
		Break	12		Pull-ups repetition rate	
	1:18				Average	0.47
		Butterfly pull-ups × 9	12	0.75	Fastest	0.54
	1:30	Round total	90	0.47	Slowest	0.35
Round 2 (15 repetitions)		Transition	4		Slope	0.00
	1:34				SD	0.11
		Thrusters × 15	30	0.50	Round repetition rate	
	2:04				Average	0.40
		Transition	8		Fastest	0.47
	2:12				Slowest	0.35
		Kipping pull-ups × 8	13	0.62	Slope	−0.05
	2:25				SD	0.06
		Break	18		Breaks	
	2:43				Count (n)	2
		Kipping pull-ups × 7	12	0.58	Total time (sec)	30
	2:55	Round total	85	0.35	Average (sec)	15
Round 3 (nine repetitions)		Transition	4		Transitions	
	2:59				Count (n)	5
		Thrusters × 9	18	0.50	Total time (sec)	32
	3:17				Average (sec)	6
		Transition	8		Down time (Breaks + Transitions)	
	3:25				Count (n)	7
		Strict pull-ups × 9	17	0.53	Total time (sec)	62
	End: 3:42	Round total	47	0.38	Average (sec)	9
		Workout total	222	0.41	Failed repetitions (n)	0

In the previous example, our 95-kg athlete completed “Fran” in 222 s (or 3:42), which equated to a repetition completion rate of 0.41 repetitions·sec^−1^. That rate provided an overall estimate of his pace but gave no indication of how pace might have differed between exercises or across rounds. Those distinctions are made clear in this latter example. Although a faster pace was seen in round 1 compared to rounds 2 and 3, thruster rate was consistent across each round (SD = 0.01) while pull-ups rate varied (SD = 0.11). Pull-ups were faster on round 1 (0.75–0.80 repetitions·sec^−1^) compared to rounds 2 (0.58–0.62 repetitions·sec^−1^) and 3 (0.53 repetitions·sec^−1^), but not when break time was considered. The athlete totaled 30 s of break time in rounds 1 and 2, which made rounds 1 (0.54 repetitions·sec^−1^) and 3 (0.53 repetitions·sec^−1^) similar and faster than round 2 (0.35 repetitions·sec^−1^). A coach or athlete might use this information to conclude that technique needed more attention than anaerobic endurance. The present example required the athlete to utilize a “butterfly” technique for round 1, a “kipping” technique for round 2, and strict pull-ups for round 3, and the athlete purposefully broke at 12 and 8 repetitions during rounds 1 and 2, respectively. These pull-up variations have been reported to alter muscle activation (36), and this example implies that repetition speed may also be different. By standardizing pull-up technique, the coach or athlete may reassess repetition speed and the impact of the strategic mid-set break on performance.

The example in Table 3 illustrates a method of organizing performance in a short-duration workout, such as “Fran,” and utilizing extracted variables (e.g., repetition rate consistency) to identify potential training targets. Although this method is suitable for any CF workout, longer workouts might require additional organization. For example, the first workout of the 2020 CFO (CFO-20.1) was a 10-round couplet of eight ground-to-overheads (men: 95 lbs., women: 65 lbs.) and 10 bar-facing burpees (3). It would be impractical to extract variables for each of the 10 rounds, analyze each round individually, and then attempt to offer recommendations for improving performance on specific rounds. Instead, dividing the workout into sections may provide more useful insight into which aspects (i.e., physiological, technical, and strategic) need attention during training. Table 4 provides an example of this for CFO-20.1, where the performances of three athletes (“A,” “B,” and “C”) are summarized over two halves of the workout, as well as overall. Data for these performances were extracted from publicly-available recordings that each athlete uploaded to the CF Leaderboard (42).

**Table 4:** Comparisons in workout and workout component completion rates between three athletes completing the first workout of the 2020 CrossFit^®^ Open.

	**Ground-to-overhead**			**Bar-facing burpees**			**Round**		
	**A**	**B**	**C**	**A**	**B**	**C**	**A**	**B**	**C**
**Repetition rate (repetitions sec** ^**−1**^ **)**									
**Average**									
Rounds 1–5	0.43	0.42	0.24	0.33	0.31	0.22	0.37	0.35	0.23
Rounds 6–10	0.41	0.34	0.21	0.32	0.32	0.25	0.35	0.32	0.23
Total	0.42	0.38	0.23	0.33	0.31	0.23	0.36	0.34	0.23
**Standard deviation**									
Rounds 1–5	0.01	0.03	0.02	0.02	0.01	0.01	0.02	0.02	0.01
Rounds 6–10	0.01	0.02	0.01	0.05	0.05	0.06	0.03	0.02	0.03
Total	0.01	0.05	0.02	0.03	0.03	0.04	0.02	0.02	0.02
**Slope**									
Rounds 1–5	0.00	−0.02	−0.01	−0.01	−0.01	−0.01	−0.01	−0.01	−0.01
Rounds 6–10	−0.01	−0.01	0.00	0.02	0.02	0.03	0.01	0.01	0.01
Total	0.00	−0.02	0.00	0.00	0.00	0.01	0.00	0.00	0.00
	**Between exercises**			**Between rounds**			**Total**		
**Down time (sec)**									
**Average**									
Rounds 1–5	1.6	1.8	4.4	1.4	3.0	7.4	3.0	4.8	11.8
Rounds 6–10	2.0	3.0	4.8	3.0	6.2	7.2	5.0	9.2	12.0
Total	1.8	2.4	4.6	2.2	4.6	7.3	4.0	7.0	11.9
**Standard deviation**									
Rounds 1–5	0.5	0.4	1.8	0.9	1.9	4.3	1.2	2.3	5.9
Rounds 6–10	0.7	1.2	1.9	0.0	1.3	1.6	0.7	1.8	3.3
Total	0.6	1.1	1.8	1.0	2.3	3.1	1.4	3.0	4.5
**Slope**									
Rounds 1–5	0.1	0.2	1.0	0.5	1.1	2.4	0.6	1.3	3.4
Rounds 6–10	0.4	0.0	−1.2	0.0	0.7	−0.7	0.4	0.7	−1.9
Total	0.1	0.2	0.0	0.3	0.7	0.2	0.4	0.9	0.2

Athletes A and B completed the workout in 9.0 and 10.1 min, respectively, whereas C only completed 175 of 180 repetitions when the 15-min time limit expired. It is immediately clear that compared to the other two athletes, C's repetition completion rate per round (and for each exercise) was slower over the entire workout and that his rest breaks (between exercises and rounds) were longer. In athlete C's case, there appears to be multiple areas in which his performance could be improved. The slower repetition pace and longer breaks suggest a need for improving aerobic and anaerobic capacity. However, his slower ground-to-overhead repetition pace may also be related to deficiencies in strength and technique. Technique during bar-facing burpees may also require attention. Thus, reexamining the video to check for technical deficiencies would be the next step before revising training to address potential physiological needs.

Analyzing the performances of A and B follows a similar process but their needs are more specific. Repetition rate (per round) was the same for both athletes over the entire workout, but average ground-to-overhead rate was lower during the second half for B. This could also have been foretold by ground-to-overhead slope for rounds 1–5, which was zero (i.e., consistent) for A and negative (i.e., slowing down) for B. Athlete A better maintained ground-to-overhead pace over the first 5 rounds and only experienced a small decline over the latter half (-0.02 repetitions·sec^−1^) whereas B's pace declined by ~0.08 repetitions·sec^−1^. Rest intervals also differed between A and B, but only by ~1.8 s over the first 5 rounds and mainly between rounds. However, over the last 5 rounds, the difference in average rest interval increased to ~4.2 s. Over the entire workout, these differences in rest equate to a 9- and 21-s difference in time devoted to transitioning. It is possible that initiating the workout with a more conservative pace would have helped B keep a more consistent pace and limit the separation seen in the second half. Endurance and technical efficiency are other factors worth consideration for B. Meanwhile, though A performed best, emphasizing a more even pace and reexamining the workout for technical errors might improve performance. Rest intervals became more consistent (from SD = 1.2 to SD = 0.7) and bar-facing burpee pace became less consistent (from SD = 0.02 to SD = 0.05).

Although analyzing video recordings of CF performance is somewhat tedious, it can provide insight on several trainable characteristics. It does not precisely quantify training workload but can provide a closer (to the cause) approximation of how an athlete handles a workload. Thus, far, this method has only been used in one study (60) and a series of poster presentations (61–64). These studies limited the analysis to repetition completion rate and quantified breaks, transitions, and failed repetitions to make comparisons among athletes and predict performance. The method provides additional opportunities to examine factors such as kinematic variation and kinetic expression amongst athletes and across a workout, but these have not yet been explored. Doing so would most likely require equipment (e.g., a high-speed motion capture system) that is not typically available to coaches and athletes, while the set up and processing would not be expeditious. Nevertheless, monitoring for changes in technique and kinetic expression might help identify inefficient movement and observe fatigue.

### Workout kinetics

Wearable technologies and linear position transducers (LPT) are commonly employed for monitoring human and barbell kinetics (65, 66), but neither have been used to quantify CF workout performance. The primary challenge is that no single technology is appropriate for all possible weightlifting, gymnastic, and monostructural exercises that might appear within a single CF workout. For instance, an LPT may be suitable whenever an exercise requires an athlete or implement (i.e., barbell) to travel a short, straight distance (e.g., weightlifting and some gymnastics) within a fixed space. The hero workout “DT” is an excellent example that meets these criteria. “DT” is a TTC workout that consists of five rounds of 12 deadlifts, nine hang power cleans, and six push jerks with 155 lbs. (70.3 kg) for men and 105 lbs. (47.6 kg) for women (30). By affixing the LPT's cord to the barbell, a coach or athlete can monitor kinetics over the entire workout without interfering with performance. This is not the case, however, when a workout requires multiple implements or transitions between modalities. In the benchmark workout “Helen,” trainees must complete 3 rounds of a 400-m run, 21 kettlebell swings (24.0 kg), and 12 pull-ups (30). Although an LPT might be suitable for measuring pull-up kinetics, and possibly hip extension kinetics during the kettle bell swing, it is useless for the 400-m run. The athlete would need to detach and reattach the cord before and after each run. One possible solution is to pair wearable technology (e.g., accelerometers, GPS) with the LPT to quantify the 400-m run, but this also introduces concerns about each technology's precision (e.g., sampling rate), validity, and reliability. Though a detailed discussion is beyond the scope of this paper, additional information one the usefulness of various technologies for quantifying performance can be found elsewhere (65, 66).

Another situation worth noting is when the workout incorporates multiple exercises that may be monitored by the same device, but not in the same exact location. “Fran” is an example where an LPT is suitable for each exercise (i.e., thrusters and pull-ups) in the workout. However, each exercise requires a different set-up and different loads to be entered into the microcomputer. Since the transducer's cord is attached to the end of the barbell for thrusters and the athlete's waist for pull-ups, two units are ideal. Expanding on the “Fran” performance described in Tables 2, 3. Table 5 provides summary kinetic data collected by two LPT's.

**Table 5:** Expressed kinetics during the benchmark workout “Fran.”

		**Distance**	**Time**	**Velocity**	**Force**	**RFD**	**Power**	**Work**
		**M**	**sec**	**m sec^−1^**	**N**	**N sec^−1^**	**W**	**N sec**
Round 1	Thrusters × 21							
	Average	1.33	0.94	1.59	683	1,389	687	909
	SD	0.03	0.04	0.07	23	127	30	38
	CV (%)	2.0	4.6	4.6	3.3	9.1	4.4	4.1
	Slope (per repetition)	0.00	0.01	−0.01	−2	−12	−4	−2
	Butterfly pull-ups × 21							
	Average	0.50	0.41	1.33	2,532	10,835	1,391	1,269
	SD	0.03	0.05	0.19	680	9,687	560	269
	CV (%)	6.6	12.5	14.6	26.8	89.4	40.2	21.2
	Slope (per repetition)	0.00	0.00	0.00	23	644	26	2
Round 2	Thrusters × 15							
	Average	1.29	0.87	1.60	685	1,394	701	884
	SD	0.00	0.07	0.11	41	181	51	53
	CV (%)	0.0	7.5	6.8	6.0	13.0	7.3	6.0
	Slope (per repetition)	0.00	0.01	−0.02	−1	−23	−9	−1
	Kipping pull-ups × 15							
	Average	0.49	0.57	1.05	1,976	4,204	1,044	959
	SD	0.04	0.12	0.23	438	1,677	222	223
	CV (%)	8.0	20.2	22.0	22.2	39.9	21.2	23.2
	Slope (per repetition)	0.00	0.01	−0.02	−13	−130	−14	−11
Round 3	Thrusters × 9							
	Average	1.33	0.96	1.55	664	1,318	669	883
	SD	0.02	0.05	0.07	48	204	32	64
	CV (%)	1.8	4.8	4.6	7.2	15.5	4.7	7.2
	Slope (per repetition)	0.00	0.01	−0.01	−7	−36	−5	−7
	Strict pull-ups × 9							
	Average	0.46	0.66	0.71	1,704	3,418	682	775
	SD	0.03	0.07	0.06	118	604	61	58
	CV (%)	6.4	11.3	8.9	6.9	17.7	9.0	7.4
	Slope (per repetition)	−0.01	0.02	−0.02	8	−178	−19	−9

Immediately, differences can be seen with the estimated work and power output described in Table 2. A greater amount of work was completed during “Fran” when measured by LPT (+4,251 J) compared to the estimate, whereas power expression was not even comparable. The estimate divides estimated work by TTC to calculate power output (2), whereas the LPT pairs measured cord velocity with estimated force to find power on each repetition. The additional precision produced average power readings that were 1.8–3.7 times greater than the estimate's calculation for the entire workout. Clearly, the estimate and LPT were not quantifying the same thing. The estimate does not account for differences in distance covered on each repetition, nor does it distinguish between “active” and “rest” time (e.g., breaks and transitions). Ignoring these severely underestimates the actual power expressed when performing an exercise. Using technology such as an LPT not only distinguishes between work and rest, but also provides a more accurate approximation of work and power expressed on each repetition of each exercise and in relation to the actual mass being moved.

The usefulness of quantifying the expression of kinetics throughout a workout extends beyond monitoring workloads. The resultant calculations provide additional insight into trainable aspects. Averaged kinetics can be used to compare rounds and athletes. For instance, average velocity decreased in round 3 for thrusters, and was markedly different among the three pull-up variations. Technique aside, differences or a decline across rounds would indicate a need to improve consistency. Calculating SD also provides a measure of consistency but here we can use it to determine whether specific rounds were more variable. For thrusters, round 2 stands out for average velocity and power, whereas patterns for other kinetics and pull-ups were not readily obvious. The exercises themselves can also be compared by calculating a coefficient of variation (CV; SD divided by mean times 100). Doing so revealed more variation in pull-ups during the first two rounds (i.e., while using a kipping or butterfly technique), and more variation in average force and rate of force development (RFD) than other kinetics. Interestingly, calculated slope was negative for nearly all kinetics for each exercise except the first round of pull-ups. Though a progressive decline in velocity, force, and power might be expected across a set due to accumulated fatigue, this did not happen. It's possible that momentum created from the butterfly technique used in round 1 facilitated greater kinetic expression. Being an example and only representative of a single athlete, it is beyond the scope of this paper to explain these observations. Rather, we aimed to simply demonstrate the utility of quantifying and examining CF workouts in this fashion.

## Limitations and future directions

Evidence on any specific topic related to acute and long-term responses to CF or high-intensity functional training is limited in quantity and generalizability. The training strategy is defined by daily workout variability (1, 2) and appropriately, workouts appearing in studies examining physiological responses have varied. This presents a major problem for making generalized conclusions about observed responses and offering recommendations for training. A meaningful, quantifiable account of the stimulus is needed to generate connections between workouts and studies, but few existing studies have provided more than basic descriptions of workout performance (e.g., repetitions completed or TTC). Retroactively applying any method discussed in this paper may not be possible, leaving the present recommendations to be considered a starting point and call for better reporting practices.

The physiological and skill-based requirements of typical CF workouts are cross-disciplinary. Athletes have been tasked with demonstrating strength and skill in performing a wide array of exercise combinations, and to sustain effort for durations that most often range between a few to 20+ min (3). Their relative strength and skill in each component, as well as the organization of tasks, will impact both the self-selected approach to each workout and the resultant physiological response. The purpose of this review was to discuss potential methods for quantifying the effort responsible for the observed response (i.e., the overall workout stress) that used to modify training. This was necessary because, although several common, objective methods for determining relative intensity and difficulty for resistance training and cardiovascular exercise exist (24, 27), they are not appropriate for quantifying CF workouts. Several are tedious are not conducive for making fair comparisons between different components that might be part of the same workout (e.g., cycling and weightlifting) or between different workout types (i.e., those scored as TTC vs. AMRAP). Several also depend on being made relative to maximal capability, which puts them in continuous need of update to account for adaptations. More importantly, their meaning is clouded by the contextual aspects of CF workouts (e.g., exercise placement and workout structure) that influence relative difficulty and the trainee's effort. By calculating repetition completion rate and/or monitoring kinetics, those aspects are better described for each possible component, as well as the overall workout. Athletes who complete assigned work more quickly or complete more work within a set duration (for a specific component or the entire workout) will perform at a faster repetition completion rate. By formatting components and workouts into the same units, comparisons between within- and between-workouts are more easily made regarding the trainee's effort in each. That said, the overall workload, exercise duration, and intensity-difficulty remain unclear and in need of some other unifying metric.

While more traditional metrics of quantifying intensity-difficulty in resistance and cardiorespiratory (24, 27) might be employed as correction factors for the repetition completion rate calculation, such a process will be limited by the potential for workouts to include gymnastic-calisthenic components. No current methods or standards have been mentioned within CF literature for quantifying the relative intensity-difficulty of the various gymnastic movements (e.g., muscle-ups and handstand walks) that might appear. It is plausible that, because skill (in the movement) heavily influences relative difficulty, scoring methods used in gymnastics competition (67) might be paired with a subjective assessment (by the coach or athlete) of skill to find relative difficulty. However, the degree of difficulty would still be affected by the specific prescription and placement within each workout, and these have yet to be explored. Nevertheless, incorporating relative component intensity-difficulty into the quantification methods described here would improve their description of context and allow for more accurate comparisons.

While quantifying each workout and associated components is useful for better understanding any observed responses and modifying training, the vastness of such as task cannot be ignored. CF workouts can involve any combination of exercises and prescriptions (1, 2). Describing physiological responses to every potential iteration is impossible and assessing the relative contribution of each associated component only further complicates the matter. Though quantifying workout and component workloads, completion rates, and/or kinetics does not overcome this challenge, their calculation provides a means to a solution. It may be hypothesized that relationships exist between workouts and their components. Currently, however, relationships among CF workouts have received very little attention (5, 11) and data regarding relationships between workout components is limited to a single abstract presentation (68). Within that presentation, average pace (repetitions·seconds^−1^·round^−1^) and a proxy of average work completed (repetitions·seconds·rounds^−1^) were calculated for each component of two CrossFit^®^ Open workouts [20.1 and 20.4, see (3) for details] and then related. The authors found that both components of 20.1 were related to only one of the components of 20.4, thus suggesting that specific workout components may act as indicators of performance in other workouts. More extensive work is clearly needed but this initial study supports using the metrics described here to quantify workout components and observe how they relate. By understanding these relationships, it may be possible to provide coaches and athletes with specific recommendations for how they might use workout components interchangeably or adjust components to increase or decrease the training stimulus.

## Conclusion

Programming for high-intensity functional training, specifically CF, varies substantially across workouts. Any workout may consist of one or more weightlifting, gymnastic, or traditional aerobic training exercises programmed at various intensities and durations. Most often, workouts receive supplementary instructions that define scoring and performance, and these usually promote high-intensity effort by maximizing density. The combination of these programming features de-emphasizes the focus on specific training targets in favor of promoting generalized adaptations across all relevant fitness domains. This characteristic is mirrored in competition, where aptitude across a wide range of physiological attributes appears to be indicative of success. The number of relevant training targets presents a potential trap for trainees because several target combinations require proper sequencing for effective and safe training. Successful athletes and coaches are seemingly able to accomplish this and avoid overtraining and injury, but their tactics remain predominantly anecdotal. Meanwhile, peer-reviewed, scientific evidence is too limited on any specific topic to allow meaningful conclusions or generalized recommendations that would help guide less experienced or successful athletes. This problem is further compounded by a lack of simple, practical, and standardized methods being available for quantifying CF workloads. Accurately quantifying the workload and difficulty of CF workouts provides coaches, athletes, and sports scientists with a practical metric to tie to any observed (or perceived) physiological response to training. Without such a metric, any changes made to programming cannot be clearly documented as being small or large in a positive or negative direction, nor can different workouts be adequately compared.

Thus, far, only a few practical methods have been suggested for quantifying CF workout performance. Aside from the inherent scoring metrics associated with any given workout (i.e., TTC or repetitions), CF-certified coaches are provided with a means for estimating work and power output that is based on an athlete's physique, prescribed loads, and duration of exercise. While the simplicity of this method has practical merit, its accuracy suffers from too many assumptions that introduce bias and imprecision across all repetitions. Pairing up this method with wearable or portable technology can improve its accuracy. However, each technology possesses its own set of inherent flaws, which may be compounded when performance cannot be measured by a single device. Alternatively, video analysis is a universal method that may be used to calculate total workout and workout component repetition completion rates, break and transition counts and durations, failed repetition counts, and subjective (or objective) ratings of technique. These metrics may be further assessed using a variety of additional calculations (e.g., by calculating averages, SD, slope) to describe their nature across an entire workout. Though more tedious than other methods described in this paper, particularly when multiple athletes must be observed, this latter method provides the most information with the least amount of error, is monetarily inexpensive, and available to anyone with access to a video recording device. Coaches and athletes are encouraged to utilize the most accurate and time efficient method at their disposal to properly monitor training and make appropriate adjustments. Meanwhile, sports scientists are encouraged to utilize one of the methods proposed in this paper when describing their sample's training habits or their study's exercise intervention to better communicate context and enable more appropriate collation of data across studies.

## Author contributions

Both authors listed have made a substantial, direct, and intellectual contribution to the work and approved it for publication.

## Conflict of interest

The authors declare that the research was conducted in the absence of any commercial or financial relationships that could be construed as a potential conflict of interest.

## Publisher's note

All claims expressed in this article are solely those of the authors and do not necessarily represent those of their affiliated organizations, or those of the publisher, the editors and the reviewers. Any product that may be evaluated in this article, or claim that may be made by its manufacturer, is not guaranteed or endorsed by the publisher.
